# Nottingham Prognostic Index in Triple-Negative Breast Cancer: a reliable prognostic tool?

**DOI:** 10.1186/1471-2407-11-299

**Published:** 2011-07-15

**Authors:** André Albergaria, Sara Ricardo, Fernanda Milanezi, Vítor Carneiro, Isabel Amendoeira, Daniella Vieira, Jorge Cameselle-Teijeiro, Fernando Schmitt

**Affiliations:** 1 Institute of Molecular Pathology and Immunology of Porto University (IPATIMUP), Porto, Portugal; 2 Institute of Biomedical Sciences of Abel Salazar (ICBAS), Porto, Portugal; 3 Department of Pathology of Hospital of Divino Espírito Santo, Ponta Delgada, Portugal; 4 Department of Pathology, Medical Faculty of University of Porto, Alameda Prof. Hernâni Monteiro, Porto, Portugal; 5 Federal University of Santa Catarina, Florianopolis, Brazil; 6 Department of Pathology, Hospital Xeral-Cíes, Vigo, Spain

## Abstract

**Background:**

A breast cancer prognostic tool should ideally be applicable to all types of invasive breast lesions. A number of studies have shown histopathological grade to be an independent prognostic factor in breast cancer, adding prognostic power to nodal stage and tumour size. The Nottingham Prognostic Index has been shown to accurately predict patient outcome in stratified groups with a follow-up period of 15 years after primary diagnosis of breast cancer. Clinically, breast tumours that lack the expression of Oestrogen Receptor, Progesterone Receptor and Human Epidermal growth factor Receptor 2 (HER2) are identified as presenting a "triple-negative" phenotype or as triple-negative breast cancers. These poor outcome tumours represent an easily recognisable prognostic group of breast cancer with aggressive behaviour that currently lack the benefit of available systemic therapy. There are conflicting results on the prevalence of lymph node metastasis at the time of diagnosis in triple-negative breast cancer patients but it is currently accepted that triple-negative breast cancer does not metastasize to axillary nodes and bones as frequently as the non-triple-negative carcinomas, favouring instead, a preferentially haematogenous spread. Hypothetically, this particular tumour dissemination pattern would impair the reliability of using Nottingham Prognostic Index as a tool for triple-negative breast cancer prognostication.

**Methods:**

The present study tested the effectiveness of the Nottingham Prognostic Index in stratifying breast cancer patients of different subtypes with special emphasis in a triple-negative breast cancer patient subset *versus *non- triple-negative breast cancer.

**Results:**

We demonstrated that besides the fact that TNBC disseminate to axillary lymph nodes as frequently as luminal or HER2 tumours, we also showed that TNBC are larger in size compared with other subtypes and almost all grade 3. Additionally, survival curves demonstrated that these prognostic factors are equally important to stratify different survival outcomes in non-TNBC as in TNBC. We also showed that the NPI retains the ability to stratify and predict survival of TNBC patients.

**Conclusion:**

The importance of this study relies on the need of prognostication improvements on TNBC, showing, at a clinical standpoint, that Nottingham Prognostic Index is as a truthful prognostic tool in TNBC.

## Background

Breast cancer comprises a complex and heterogeneous group of diseases at clinical, morphological and molecular levels [1-3]. It is clear that breast tumours of the same histological type show remarkably different clinical behaviour, which is probably a reflex of their distinct pattern of molecular aberrations [1,4]. Microarray technology has changed the way we understand breast cancer classification by looking towards a molecular-based approach instead the traditional morphology and histopathological-based system [5,6]. Pioneered by the Stanford group [6-9] and lately explored by several other groups, a new taxonomy for breast cancer based on expression profile has claimed that the morphological heterogeneity of breast cancer can be recapitulated and systematically classified at the transcriptomic level and into clinically meaningful groups [10-12]. Such studies have shown that the molecular profile of breast cancer present a systematic variation which allowed its differential identification into two distinct branches [11], the ER-positive branch, comprising the luminal A and B subtypes, and three ER-negative branch, which comprises at least, two reproducible subtypes: the HER2-overexpressing group and the basal-like group [6,8,9,13-15]. An additional group of tumours displaying molecular features of normal breast tissue, and therefore named as "normal-like", has been also included in this ER-negative branch. However, it has been suggested that this group represents an artefact with high contamination from normal breast tissue rather than a distinct molecular subtype [14]. Among the molecular subtypes of breast cancer identified through gene expression profiling studies, none has generated as much interest or controversy as the basal-like breast cancer group (BBC) [1]. Recently, a review published by a large group of renown breast pathologists and clinicians, advocated that there is still no internationally accepted definition for BBC and discussed how best to define these tumours [1]. Nevertheless, it is commonly accepted that the term "basal-like" reflects the similarity of the protein expression profile of these tumours with the one of basal epithelial cells of the normal mammary gland [16-19], including high-molecular-weight cytokeratins (CK) 5/6, 14 and CK17, vimentin, P-cadherin, caveolins-1 and 2, αB-crystalin and fascin [8,20-29]. BBC, the only group consistently defined by gene expression arrays [30], account for up to 15% of all breast cancers [1,11]. These tumours frequently lack or show low levels of ER and PR, lack HER2 overexpression and amplification [21,31,32] and in approximately 85% of the cases display p53 expression by immunohistochemistry or TP53 mutations [8,33]. Additionally, BBCs show exceedingly high levels of proliferation-related genes [6,8,9,13] and express EGFR in a significant number of cases [21,34]. Defined by microarray-based expression profiling or by panels of immunohistochemical markers as surrogates, BBCs are known by their clinically aggressive behaviour [8,10,21,32].

Specimens that display BBC features (hormone receptors and HER-2 lack of expression), are called, in routine practice, as "triple-negative" breast cancer (TNBC). Controversial and provocative data has been recently published questioning whether TNBC and BBC are synonymous. Because a majority of BBCs are also TNBCs and approximately 80% of TNBCs are also BBCs [21,35], it has been claimed that the TNBC and BBC are effectively synonymous [36,37]. However, clinical, microarray and immunohistochemical data have shown that equating TNBC with BBC is misleading [15,33].

TNBC patients lack the benefit of routinely available target therapy, which explains the undeniable growing attention of both pathologists and oncologists as an easily recognisable group of breast cancer with aggressive behaviour and poor therapeutic options [2,38]. The prognosis of women with TNBC is significantly poor, compared to women with other subtypes of breast cancer. The higher recurrence and mortality rates of TNBC patients may be in part explained by different routes of metastatic spread [39]. There are conflicting results on the prevalence of lymph node metastasis at the time of diagnosis in TNBC patients [11]. Some studies described a higher prevalence of lymph node metastasis in TNBC [40], while others have found no statistical differences [3] or even an inverse association between TNBC and lymph node metastasis [41]. The currently accepted theory is that TNBCs seems to disseminate to axillary nodes and bones less frequently than the non-triple-negative cancers, presenting a preferential haematogenous route [32,42-44] with a proclivity to develop metastatic deposits in the brain and lungs [11].

The Nottingham Prognostic Index (NPI) combines nodal status, tumour size and histological grade [45], reflecting metastatic behaviour, growth rate and genetic instability of breast cancers [46,47]. Most importantly, as a continuous variable, NPI offers a responsive and sensitive means of modelling a *continuum *of clinical aggressiveness [46], indexing the outcome likelihood of invasive breast cancer patients [48]. NPI can define 3 subsets of patients with different probabilities of dying from breast cancer; good (≤3.4), moderate (3.41 - 5.4), and poor (> 5.4) prognosis groups [48]. Three factors, found to be independently associated with survival on multivariate analysis, were combined to give the NPI algorithm. One of these factors is the lymph node stage, which has traditionally been regarded as the most powerful prognostic factor in breast cancer. The greater the number of nodes involved, the worse the prognosis [45].

As above mentioned, TNBCs are believed to infrequently disseminate to axillary lymph nodes in favour of distant and visceral metastatic spread [32,39,42], an assumption that theoretically jeopardize the reliability of using NPI as a tool for TNBCs prognostication, since lymph nodal status is a major component for NPI calculation.

Herein, we investigated a large series of breast tumours and also a second cohort only composed by TNBC phenotype. These cohorts were used to test the clinical utility of NPI in predicting breast cancer patient outcome. Comparative analyses were performed within the TNBC subgroup of patients in order to evaluate the contribution of each Nottingham Prognostic components to the risk of worse survival and prognosis in TNBCs.

## Methods

### Patient Selection

A series of 467 primary invasive breast carcinomas diagnosed between 1978 and 1992 were retrieved from the Pathology Department, Hospital Xeral-Cíes, Vigo, Spain. Patients' ages ranged from 28 to 92 years old, mostly submitted to therapeutic surgery and/or surgery plus chemotherapy in the case of lymph node-positive patients. The formalin-fixed paraffin-embedded histological sections were reviewed and the diagnoses confirmed by two trained pathologists (FS and FM). The tumours were characterized for clinical and pathological parameters - namely age, tumour size, lymph-node status, and histological grade (Table 1). Whenever was possible, NPI was calculated for each of the patients by using the following equation: NPI = 0.2 × tumour size (cm) + grade (1-3) + lymph node status (1-3) [47]. Patient follow-up information was available for 455 cases, ranging from a 1 to 120 months after the diagnosis. Overall and disease-free survival time was defined as the time from the date of surgery to the date of death or to the date of breast cancer derived relapse/metastasis, respectively.

**Table 1:** Patients characteristics and tumour parameters

Variable (*N *= 467)	Data
**Age at Diagnosis, years**	
Mean and standard deviation	59 ± 13
Range	64 (Min 28; Max 92)
**Tumour Size (cm)**	
Mean and standard deviation	3.110 cm ± 2.00 cm
Range	15.6 (Min 0.4; max 16)
T1: < 2 cm	101 (24.7%)
T2: 2-5 cm	245 (59.9%)
T3: > 5 cm	63 (15.4%)
Not assessed	58
**Lymph Node Invasion**	
Present	207 (56.6%)
Absent	159 (43.4%)
Not assessed	101
**Histological Grade**	
Grade I	81 (18.3%)
Grade II	135 (30.5%)
Grade III	227 (51.2%)
Not assessed	24
**Oestrogen receptor**	
Positive	309 (66.5%)
Negative	156 (33.5%)
Not assessed	2
**Progesterone Receptor**	
Positive	228 (48.9%)
Negative	238 (51.1%)
Not assessed	1
**HER2**	
Positive	68 (14.7%)
Negative	395 (85.3%)
Not assessed	4
**Nottingham Prognostic Index**	
NPI < 3.4	99 (24.4%)
3.4 ≤ NPI ≤ 5.4	188 (46.4%)
NPI > 5.4	118 (29.2%)
Not assessed	62
**Molecular Subtype**	
Luminal	343 (73.6%)
HER2 Over-expressing	33 (7.1%)
Triple Negative	90 (19.3%)

An additional cohort of 168 TNBC patients was included in this study and analysed separately. This cohort resulted from the combination of the 89 triple negative tumours from the former series, plus 25 cases from the Divino Espirito Santo Hospital (Ponta Delgada-Portugal), 29 cases from the Federal University of Santa Catarina (Florianopolis-Brazil) and 25 cases from the São João Hospital (Porto-Portugal). All these TNBC specimens were evaluated and classified accordingly with the same criteria and by the same panel of pathologists. Patients followed the same therapeutic regimen design as the general series.

This study was conducted under the national regulative law for the handling of biological specimens from tumour banks, being the samples exclusively available for research purposes in retrospective studies.

### Tissue microarray construction and immunohistochemistry

Representative tumour areas were selected on haematoxylin-eosin-stained sections. At least two tissue cores (0.6 mm in diameter) were obtained from each selected specimen and deposited into a recipient paraffin block, using a tissue microarray (TMA) workstation (*Manual Tissue Arrayer, Beecher Instruments, Inc*.). The TMA blocks were designed and built as previously described [20] and non-neoplastic tissue cores were included as controls.

In order to classify all breast cancer tumours according with the molecular subtype, immunohistochemistry was performed and the expression of breast cancer biomarkers [49], namely the hormonal receptors ER and PR, the tyrosine kinase receptors HER2 and EGFR, the basal cytokeratins CK5 and CK14, and also P-cadherin and vimentin were evaluated. Immunohistochemical expression was detected using HRP polymer (Cytomation Envision System HRP, DAKO, Carpinteria, CA), according with the manufacturer's instructions. Both methods used diaminobenzidine as chromogen.

### Immunohistochemical evaluation

The expression of ER, PR, HER2, EGFR, CK5, CK14, P-cadherin and vimentin was evaluated according with the grading systems previously described [49]. These immunohistochemical results were used to classify the tumours into the different breast cancer subtypes, namely Luminal, HER2-OE and Triple-negative (TNBC).

### Statistical analysis

Statistical analysis was performed using SPSS statistics 17.0 software (SPSS Inc., Chicago, IL, USA). Continuous variables were presented as the mean standard deviation, and categorical variables were presented as number (percentage). Mean differences for continuous variables such as tumour size was performed using unpaired T-test with a 95% confidence interval. Association between different expression subtypes and the clinicopathological features were assessed by Pearson correlation and chi-squared tests.

Survival curves were estimated by the Kaplan-Meier method using the long-rank test to assess significant differences in survival. Cox regression models were fitted to estimate hazard ratios [(HR, 95% confidence interval (CI))] for the classical prognostic factors that constitute the Nottingham Prognostic Index, namely, tumour size, tumour grade and lymph-node status (LNS). Similarly, Cox regression was used to calculate HR for the NPI scoring in different expression subtypes. For all analysis, a significant level of 5% was considered.

## Results

### NPI as a predictor of survival in breast cancer subtypes

**Figure 1:**
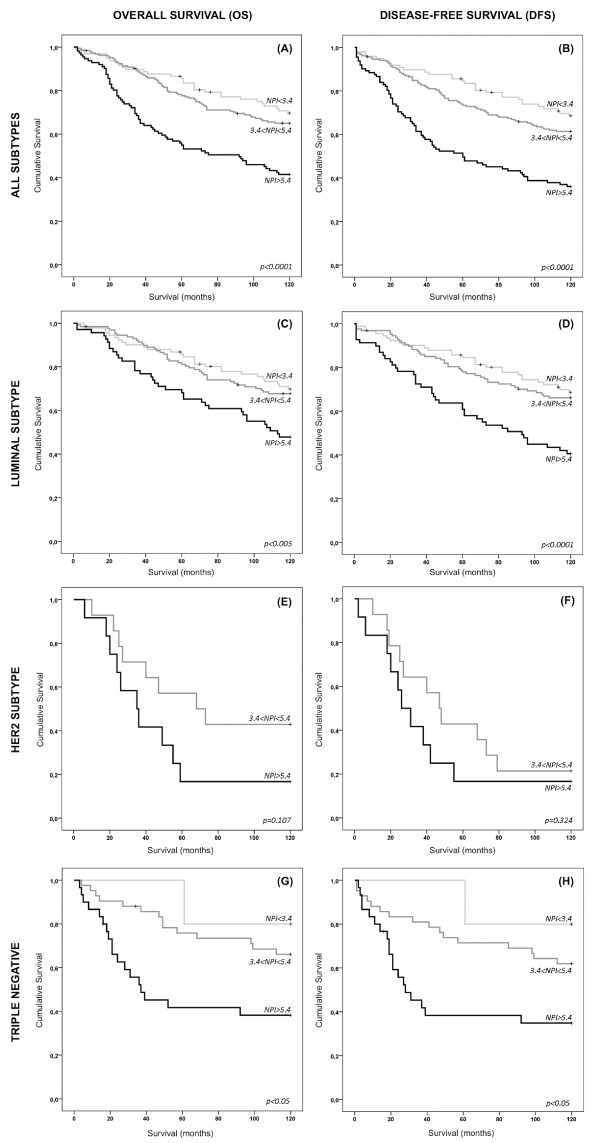
**Long term overall and disease-free survival in breast cancer subtypes according with Nottingham Prognostic Index**. Kaplan-Meier plots for a general series comprising all subtypes of invasive breast tumours (**A **and **B**). As expected, NPI clearly separate different groups with distinct outcomes, showing that patients with NPI > 5.4 presented a much worse prognosis compared with patients with moderate and good prognosis; (**C **and **D**) Kaplan-Meier plots for luminal subtype breast carcinomas. NPI is similarly able to stratify breast cancer patient into different survival outcomes; (**E **and **F**) Kaplan-Meier plots for HER2 subtype breast carcinomas. None of the patients encompassing this subgroup presented a NPI < 3.4. Although lacking statistical association, NPI is still able to discriminate between moderate and poor prognosis patients; (**G **and **H**) Kaplan-Meier survival plots for TNBC subtype. Most of the patients within this subtype fell into the group with NPI > 5.4. Even though, NPI had the statistically significant power to stratify patients with distinct outcomes.

Using the follow-up survival data from 455 breast cancer patients, we stratified the breast-cancer-specific outcome, according with the NPI status. The NPI ranged from 2-8.4 (mean 4.8). In the whole series, and in line with what has been shown, NPI was able to stratify breast cancer patients into good, moderate and worse prognosis in terms of overall and disease-free survival (Figure 1A and 1B, respectively). The outcome stratification ability of NPI was also tested in specific breast cancer expression profiles. The same strength of statistical significance was also observed in the subset of luminal tumours (Figure 1C and 1D). An interesting finding was the one observed in the HER2-OE subgroup, where none of the cases was scored as NPI < 3.4, reinforcing the poor prognosis of this breast cancer subtype. Here, although the Kaplan-Meier curves showed a clear difference between the survival of NPI > 5.4 and 3.4 < NPI < 5.4 patients, especially concerning overall-survival, the statistical value was not significant (Figure 1E and 1F). In the cohort of TNBC, unequivocal patient outcome stratification was draw by the NPI status. Notably, only one case was scored with NPI < 3.4, confirming the aggressive behaviour pattern of these tumours. In fact, in both overall and disease-free survival Kaplan-Meier curves (Figure 1G and 1H), TNBC patients with NPI > 5.4 are clearly separated from the good/moderate outcome curve, reinforcing not only the worse survival of the high-scored-NPI TNBC patients, but also suggesting the value of NPI as a predictor of survival in TNBC patients.

### Tumour size, histological grade and LNS in Luminal, HER2-OE and TN breast cancer

Each clinical-pathological feature that constitutes the NPI equation was explored in the context of luminal, HER2-OE and TNBC groups. The mean tumour size was calculated for the three breast cancer subgroups. As shown in Table 2 the mean tumour size of TNBC was visibly higher compared to the average size of HER2-OE and luminal tumours, which displayed the lowest mean size. Using unpaired comparison analysis (T-test, 95% CI), to evaluate the significance of the difference found in tumour size between TNBC and each of the other subtypes, we showed that tumour size of TNBC was statistically different from the luminal subtype (p < 0.001) and from the HER2-OE subtype (p = 0.05). Concerning histological grade, Chi-square test showed that there was a significant association between grade and the different molecular subtypes (p < 0.0001). The Table 2 shows that 78% and 71% of the HER2-OE and TNBC patients, respectively, were high-grade tumours, while the frequency distribution of tumour grade among luminal subtype tumours was rather homogeneous. Interestingly, a non-statistically significant association between LNS and the different expression subtypes was observed, showing that the occurrence of lymph-node metastization is a similarly frequent event across all the molecular subtypes, but most importantly, that the extension of these lymph-node involvement is as relevant in TNBC tumours (26.4%) as it is for luminal (29.4%) or HER2-OE breast cancers (33.3%) (Table 2).

**Table 2:** Nottingham Prognostic Index components on triple-negative and non-triple-negative breast cancer subtypes

	Nottingham Prognostic Index Components								
Molecular Subtype	Tumour Size Mean ± St. Error			Tumour Grade			Lymph Node Status		
				I	II	III	None	1 < LNS < 3	LNS > 3
**Luminal**	2.92 cm ± 0.105			24.1%	38%	38%	43.9%	26.7%	29.4%
	*(N = 302)*			*(N = 324)*			*(N = 262)*		
**HER-OE**	3.08 cm ± 0.203			*4.9%*	*16.5%*	*78.6%*	*42.4%*	*24.2%*	*33.3%*
	*(N = 93)*			*(N = 103)*			*(N = 99)*		
**Triple Negative (TNBC)**	3.70 cm ± 0.211			4.9%	23.9%	71.2%	48.6%	25%	26.4%
	*(N = 155)*			*(N = 163)*			*(N = 148)*		

		Luminal	*P*-value*						
**Statistics**	TNBC* vs*		p < 0.001	*P*-value** < 0.0001			*P*-value** Not significant		
		HER2-OE	P = 0.05						

*ANOVA was used to compare the means of the three groups

** *P*-values were calculated with the use of the χ^2 ^test

### High-scored-NPI lesions and its relation with tumour size, grade and LNS in a subset of TNBC

Using only the cohort of 164 TNBC patients, we evaluated the association of each of the Nottingham Prognostic components to the NPI augmentation. A boxplot graphic (Additional file 1) was draw to show the significant association of tumour size, histological grade and lymph node status to high scores of NPI in TNBC. Using Chi-square test we observed a strong association between larger tumours (p < 0.0001), displaying high histological grade (p < 0.0001) and with extensive lymph node invasion (p < 0.0001), with the worst outcome group, represented by NPI > 5.4 (Additional file 1). Besides the evidence that nearly 72% of TNBC are grade III tumours, therefore clearly contributing for a high NPI, it is however important to stress that the contribution of LNS also clearly associates with high NPI. Moreover, similarly with what was shown for tumour larger than 5 cm, all the TNBC with more than 3 metastatic lymph nodes presented a NPI > 5.4 (Additional file 1), showing that LNS is a determinant factor to predict worse prognosis in TNBC patients.

Tumour size is theoretically associated with the increased likelihood of lymph node invasion in breast cancer. In fact, we demonstrated that in non-TNBC there was a strong association (p < 0.0001) between tumour size and LNS, where 47% of patients with tumours larger than 5 cm presented extensive metastization (Table 3). In TNBC patients, 44% of patients with larger tumours also showed a significant trend (p < 0.001) to display more extensive lymph node invasion (Table 3). An additional analysis was also performed considering the presence or absence of lymph nodes involved, and herein, we observed that 61% of TNBCs with sizes < 2 cm lacked lymph node involvement, whereas approximately 78% of TNBCs with sizes > 5 cm displayed axillary lymph node invasion. These results showed that larger tumours frequently metastasize to lymph nodes, either being non-triple negative or TNBC lesions.

**Table 3:** Association of tumour size and lymph node status in triple-negative and non-triple-negative breast cancer

	Non-Triple Negative Breast Cancer			Triple Negative Breast Cancer		
**LNS**	**None**	**1 < LNS < 3**	**LNS > 3**	**None**	**1 < LNS < 3**	**LNS > 3**
**TS**						

**Tumour Size < 2 cm**	66.7%	22.7%	10.7%	60.8%	32.1%	7.1%
**Tumour Size 2-5 cm**	*39.4%*	*26.1%*	*34.5%*	*56.7%*	*19.8%*	*23.5%*
**Tumour Size > 5 cm**	19.4%	33.3%	47.2%	22.2%	33.3%	44.5%

**Statistics**	*(N = 276)*	*P*-value* < 0.0001		*(N = 145)*	*P*-value* < 0.001	

### Significance of NPI components to breast cancer survival and mortality risk in TNBC patients

**Figure 2:**
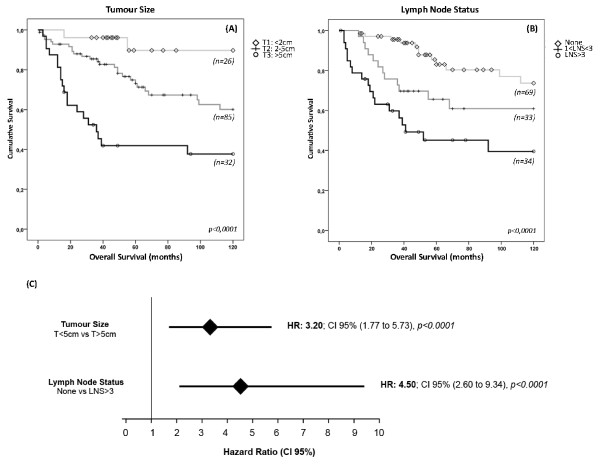
**Significance of the NPI components to breast cancer mortality risk and survival in TNBC patients**. Kaplan-Meier survival curves for tumour size (**A**) and for lymph node status (**B**). Both NPI components are highly statistically relevant to predict long term survival in TNBC patients; (**C**) Hazard ratio (CI 95%) in the TNBC cohort was calculated for tumour size and lymph node status. Patients with triple-negative tumours larger than 5 cm display a 3.2-fold risk of death by breast cancer; Concerning axillary lymph node status, TNBC patients with lymphatic involvement higher than 3 nodes have a 4.5-fold risk of breast-cancer specific death.

To evaluate the relevance of each NPI component to the survival of TNBC patients we used the follow-up data available for the TNBC cohort and survival curves were estimated by the Kaplan-Meier method. Survival curves demonstrated that TNBC patients with larger breast tumours showed a significant difference towards worse survival time (p < 0.0001; Figure 2A). Similarly, TNBC patient survival is seriously affected by the lymph node status (p < 0.0001; Figure 2B).

As previously demonstrated by univariate hazard analysis [50], we also showed that tumour size, histological grade and lymph node status were significant predictors of overall survival in breast cancer series. The same Cox proportional analysis was used to estimate the risk associated to the survival difference found by Kaplan-Meier curves in TNBC cohort. We observed that tumour size was a significant predictor of survival in this subset of tumours, showing that patients with larger tumours carry about 3.2-fold-increased risk of breast cancer-related death (HR = 3.20, 95% CI: 1.77 to 5.73), compared to breast cancer patients with tumours smaller than 5 cm (Figure 2C). Notably, when risk analysis were applied to lymph node status, we observed that TNBC patients with more extensive lymph node invasion, hold approximately 4.5-fold-increased risk of breast-cancer-related death (HR = 4.50, 95% CI: 2.16 to 9.34), compared to patients lacking axillary invasion (Figure 2C).

## Discussion

The prognosis of women with TNBC is significantly poor, compared to women with other subtypes of breast cancer. The underlying difference in recurrence and patient mortality rates may be explained in part by different routes of metastatic spread [51]. The current theory point out to the suggestion that TNBCs metastasize to axillary nodes and bones less frequently than the non-triple-negative subset of breast tumours, favouring a haematogenous spread [32,42-44].

In terms of survival, it has been described that the survival curve shape for TNBC or BBC differs from that of patients with other types of breast cancer: there is a sharp decrease in survival during the first 3 to 5 years after diagnosis, but distant relapse after this time is much less common [31,40,41,52]. In a study published by Dent and colleagues, the median time to death was 3.5 years for TNBC compared to 5.7 years for patients with other cancers [39]. In fact, as we can infer by the survival functions, TNBC experienced a severe decrease in their outcome before 48 months, a curve shape that overlaps with the one draw by the NPI in those patients. These findings reinforce the reliability of NPI as a tool to be reproducibly used in TNBC tumours.

In our cohort of TNBC we have found that tumour size is considerably higher compared with other subtypes of breast cancer. This difference in tumours size was strongly significant in relation to luminal, but not so marked when compared with HER2 tumours. Additionally, we found that, histologically, most TNBCs were high grade tumours. These results concerning tumour size and grade are largely in accordance with previous studies where these prognostic factors were studied within breast cancer subtypes, with special emphasis on TNBC *versus *non-TNBC patients [40,50,53,54]. One of these studies used a notably large series of TNBC (6.370 patients) and non-TNBC (44.704 patients), and similar findings concerning histological grade and tumour size were found [53]. Interestingly, we found no difference regarding lymph node metastization between luminal, HER2-overexpressing tumours and TNBC. Thus, although it has been suggested that TNBC tend to disseminate in a lower frequency to lymph nodes, we found that 51.4% of TNBC developed metastasis to lymph nodes. This percentage shows that 1) the lymph node involvement in TNBC is as frequent as in other subtypes of breast cancer and, 2) the extension of this involvement do not differ between breast tumour subtypes. In the last 4 years, interesting studies have reported data concerning positive lymph node status in TNBC compared to non-TNBC, describing percentages of positive lymph nodes in TNBC ranging from 42.5% [55] to 54.4% [40], therefore, corroborating the results presented herein. Based on that, and even considering some putatively less prominent lymph node involvement in TNBC, which accordingly to our results was not observed, tumour size and grade variables are someway playing a compensatory score augmentation to NPI algorithm. Additionally, survival curves concerning tumour size and lymph node status demonstrated that these prognostic factors are equally important to stratify survival outcomes in non-TNBC as in TNBC. Taken together, and considering that the majority of TNBC lesions are grade III, these largely studied prognostic factors are reliable to be used in the assessment of NPI in TNBC. Interestingly, we also found a slight association between tumour size and lymph node status in TNBC. This result contributes to some controversy concerning the existence or not of a relationship between size and lymph node status, since some authors already argued a lack of association between these two prognostic factors in TNBC [40]. In our TNBC series, we have a proportion of 74% of BBC. The discrepancies about the association between tumour size and lymph node status could be explained if the TNBC cohort used by Dent *et al*. were enriched in basal-like breast tumours compared with the cohort we studied here. These hypothesis lay on a robust study recently published by Ellis group, where although a trend (non-significant) to display lymph node involvement with increasing tumour size was seen (especially in tumours larger than 4 cm), basal-like tumours do not seem to obey the "size-node" rule [56].

## Conclusions

While basal-like tumours is a designation only revealed by gene profiling signatures [6], TNBC is a clinico-pathological classification which identifies a group of breast cancer with aggressive behaviour [2,38], and where improvements on therapy development and prognostication are compulsory. In the study presented herein, we demonstrated that besides the fact that TNBC disseminate to axillary lymph nodes as frequently as luminal or HER2 tumours, we also showed that TNBC are larger in size compared with other subtypes and almost all grade III, therefore making truthful, at a clinical standpoint, the applicability of NPI as a prognostic tool in TNBC.

## Competing interests

The authors declare that they have no competing interests.

## Authors' contributions

AA carried out all the statistical studies, participated in the coordination of the study, intellectual content and drafted the manuscript. SR participated Pathologists VC, IA, DV, FM and JCT helped in the acquisition of data and evaluated the results of the clinic-pathological analysis. SR and FM were involved in critical revising and for important intellectual content. JCT and FS participate in the design of the study and coordination of the manuscript. All authors read and approved the manuscript.

## Pre-publication history

The pre-publication history for this paper can be accessed here:

http://www.biomedcentral.com/1471-2407/11/299/prepub

## Supplementary Material

Additional file 1 **Association of tumour size (A), histological grade (B) and lymph node status (C) to high scores of NPI in TNBC**. The boxplot graphic show an association between larger tumours, displaying high histological grade and with extensive lymph node invasion, with tumours clustered into the worst outcome group, represented by NPI > 5.4 (p < 0.0001) (**A**, **B **and **C**). The graphic highlights the contribution of lymph node status to the augmentation of NPI, showing that LNS is a determinant factor to predict worse prognosis in TNBC patients. Click here for file
