# Supplementary material for: Nottingham Prognostic Index in Triple-Negative Breast Cancer: a reliable prognostic tool?
Source: BMC Cancer. 2011 Jul 15;11:299. doi: 10.1186/1471-2407-11-299 (PMC3151231; doi:10.1186/1471-2407-11-299)

Nottingham Prognostic Index in Triple Negative Breast Cancer: A Reliable Prognostic Tool?

Breast Cancer Research and Treatment

André Albergaria, Sara Ricardo, Fernanda Milanezi, Vítor Carneiro, Isabel Amendoeira, Daniella Vieira, Jorge Cameselle-Teijeiro, Fernando Schmitt
Corresponding Author: Fernando Schmitt, IPATIMUP, fschmitt@ipatimup.pt

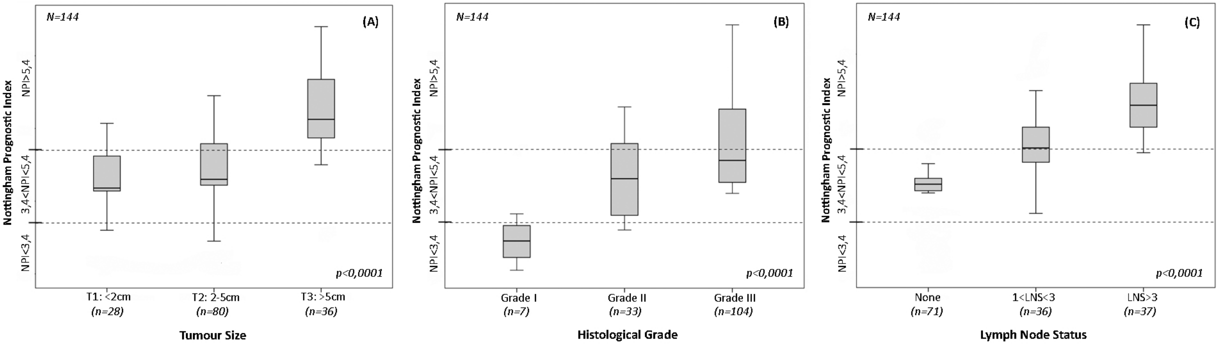

Supplement: Additional file 1 — Association of tumour size (A), histological grade (B) and lymph node status (C) to high scores of NPI in TNBC. The boxplot graphic show an association between larger tumours, displaying high histological grade and with extensive lymph node invasion, with tumours clustered into the worst outcome group, represented by NPI > 5.4 (p < 0.0001) (A, B and C). The graphic highlights the contribution of lymph node status to the augmentation of NPI, showing that LNS is a determinant factor to predict worse prognosis in TNBC patients. [file 1471-2407-11-299-S1.PDF]
